# Dual trajectories of short-term and long-term sickness absence and their social- and health-related determinants among women in the public sector

**DOI:** 10.1093/eurpub/ckae023

**Published:** 2024-02-20

**Authors:** Johanna Suur-Uski, Pi Fagerlund, Hanna Granroth-Wilding, Aino Salonsalmi, Ossi Rahkonen, Tea Lallukka

**Affiliations:** Department of Public Health, University of Helsinki, Helsinki, Finland; Department of Public Health, University of Helsinki, Helsinki, Finland; Biostatistics Consulting Service, Medical Faculty, University of Helsinki, Helsinki, Finland; Department of Public Health, University of Helsinki, Helsinki, Finland; Department of Public Health, University of Helsinki, Helsinki, Finland; Department of Public Health, University of Helsinki, Helsinki, Finland

## Abstract

**Background:**

Short- and long-term sickness absence (SA) vary in their determinants. We examined short- and long-term SA contemporaneously as two interconnected phenomena to characterize their temporal development, and to identify employees with increasing SA at an early stage.

**Methods:**

We extracted 46- to 55-year-old employed women from the Helsinki Health Study occupational cohort during 2000–17 (*N* = 3206) and examined the development of short- (1–14 days) and long-term (>14 days) SA using group-based dual trajectory modelling. In addition, we investigated the associations of social-, work- and health-related factors with trajectory group membership.

**Results:**

For short-term SA, we selected a three-group solution: ‘no short-term SA’ (50%), ‘low frequency short-term SA’ (40%), and ‘high frequency short-term SA’ (10%) (7 spells/year). For long-term SA, we also selected three trajectory groups: ‘no long-term SA’ (65%), ‘low long-term SA’ (27%), and ‘high long-term SA’ (8%). No SA in the short-term SA model, indicated a high probability of no SA in the long-term model and vice versa. The developmental pattern was far less certain if participant was assigned to a trajectory of high SA in either one of the models (short- or long-term SA model). Low occupational class and poor health behaviours were associated with the trajectory groups with more SA.

**Conclusion:**

SA does not increase with age among most employees. If either SA rate was high, the developmental patterns were heterogenous. Employers’ attention to health behaviours might aid in reducing both short- and long-term SA.

## Introduction

Sickness absence (SA) allows employees to recuperate, but simultaneously places a financial burden on society, with relatively high SA rates in Europe.^1^ The determinants behind SA are multifaceted; in addition to socioeconomic, health-, and work-related factors, such as occupational demands,^2–6^ there is variation associated with political changes in SA benefits, the economy and perceived job security.^7^^,^^8^

Short-term SA (1–14 days) is mainly considered to be absence caused by minor infectious diseases and indolent conditions.^9^ Contrary to this, studies on municipal employees have identified that earlier short-term SA precedes long-term SA^10^^,^^11^ and permanent work disability.^12^ It has also been proposed as a coping behaviour to deal with work-related strain.^6^

Long-term SA (>14 days) is a well-documented indicator of poor health.^13–16^ Multiple health behaviours,^3^^,^^17^ socioeconomic factors^2^^,^^18^ and work environment factors^4^ are widely studied risk factors of long-term SA. Older workers and women have more long-term SA,^5^^,^^19^ but this simplification does not fully represent the variation within these groups. Our previous study on the developmental patterns of long-term SA identified three distinct trajectory groups among 50 to 60-year-old municipal employees.^20^

Despite their intertwined relationship, short- and long-term SA are often studied with short-term SA as the predictor and long-term SA^21^ as the outcome and only few studies have analysed their coexistence and development. We consider short- and long-term SA as coexisting but linked phenomena that reflect underlying characteristics of work ability with potentially differing predictors. To model this, we utilize group-based dual trajectory modelling^22^ which allows the joint analysis of two separate outcomes without combining them into a single measurement. With dual trajectory analysis an elaborate picture of the connections between trajectory groups can be given. For example, a customary interpretation of a correlation coefficient is that it applies equally to all participants, while alternatively, for some subpopulations the association might be little and for others larger, which can be examined with dual trajectory analysis. Further, examination of the trajectory groups determinants enables studying differences between occupational classes and health outcomes. An analysis of the contemporaneous occurrence and development of these two SA outcomes could facilitate better understanding of SA as a phenomenon. To the best of our knowledge, this is the first study employing dual trajectory modelling in studying short- and long-term SA.

In this study, we examined the dual developmental trajectories of short- and long-term SA among women working in the public sector and considered the trajectories’ connections with a range of social-, work-, and health-related factors.

## Methods

Our study is part of the ongoing Helsinki Health Study (HHS) that examines health and well-being of the employees of the City of Helsinki, Finland. The HHS cohort consists of employees of the City of Helsinki who turned 40, 45, 50, 55 and 60 years old at study baseline during years 2000–02 (*N* = 8960, response rate 67%, 80% women).^23^ Follow-up questionnaires were mailed in 2007 (response rate 83%), 2012 (response rate 79%) and 2017 (response rate 82%).

### Study population

We extracted a subsample of the HHS cohort with informed consent to link their survey data to the register comprising information on SA and retirement. We included only women and only years during which their employment contract lasted through the year for the reliability of SA information (*N* = 5004). We focused on women firstly because most public sector employees in Finland are women and our cohort corresponds to that. Secondly, the largest employment sectors—healthcare and social—are dominated by women.

Based on our previous findings,^20^ we included employees who were 46–55 years old during years 2000–17 (*N* = 4249, excluding *N* = 755). Participants’ SA data were not considered after retirement (excluding *N* = 55). We further required three years follow-up on SA information either separately or continuously (excluding *N* = 988). In effect, the minimum follow-up was 3 years, and the maximum 10, with a mean follow-up time of 8.5 years. The final analytical sample size was 3206.

### Sickness absence data

The SA information was derived from the City of Helsinki personnel register. We used two outcomes: short-term SA (1–14 days) and long-term SA (>14 days).

The outcomes were the annual number of short-term SA spells and the annual number of long-term SA months. We modelled short-term SA using spells instead of cumulative short-term SA days, since previous research suggests that work disability is initially marked by recurring short-term SA spells.^10^^,^^11^^,^^24^ Long-term SA days per year were transformed to account for months due to analytical purposes as follows: 0–13 days = 0 months, 14–29 days = 1 month, 30–59 days = 2 months, etc., and 12 months = 330 or more SA days.

### Sociodemographic and socioeconomic factors

Age and marital status were gathered from the baseline questionnaire. We dichotomized marital status as cohabiting (married/cohabiting) or non-cohabiting (divorced/widowed/single). Occupational class was derived from the employer’s personnel register at baseline and classified as follows: professionals and managers (such as teachers, doctors, managers with subordinates), semi-professionals (such as nurses, foremen), and routine non-manual and manual employees, which combines occupational classes routine non-manual employees (such as child minders, assistant nurses) and manual workers (such as transport workers, cleaners).^25^ We combined routine non-manual and manual employees because the most common occupations in both groups share a similar characteristic (physically demanding job).

### Work-related factors

We extracted information on the employment sector from the employer’s personnel register. Four occupational sectors were formed: (i) healthcare (primary healthcare, hospital services), (ii) social (social welfare services, elderly care), (iii) education (primary and secondary school, vocational training) and (iv) other sectors (culture, leisure, rescue, financial administration, transport).

Other work-related factors were gathered from the questionnaires. Work was categorized as regular daytime work (including daytime work with on-call nightshifts) or shift work (including shift work with regular night shifts, regular night work, and work type other). Work–home satisfaction was asked by the question ‘How satisfied are you with combining paid work and family?’. Replying satisfied or somewhat satisfied was classified as (work–home satisfaction) ‘satisfied’ and replying somewhat not satisfied or not satisfied as ‘dissatisfied’.

### Health-related factors

Smoking was dichotomized into non-smoking (including ex-smoking) and smoking (current smoking, daily or occasionally). Body mass index (BMI) was calculated from self-reported height and weight and categorized into healthy weight (BMI <25), overweight (25≤BMI< 30), and obesity (BMI ≥ 30). Leisure-time physical activity was estimated using metabolic equivalent (MET) hours^26^ and divided into: low (<14 MET-h/week), intermediate (≥14 MET-h/week with only moderate activity) and high (≥14 MET-h/week including vigorous activity). The Jenkins four-item questionnaire was used to indicate sleep problems.^27^^,^^28^ If at least 1 of the 4 symptoms occurred at least 15 times within the past 4 weeks, the participant was categorized as having sleep problems. Covariates were derived from all the questionnaires and the mode was used to determine the most common answer for each participant.

### Statistical methods

Group-based dual trajectory modelling utilizes a group-based trajectory model approach which assumes that the population is formed by separate subpopulations (trajectory groups),^22^ but instead of studying one outcome, it models two outcomes that are distinct but related in their development.

The dual trajectory model estimates three main parameters to describe individuals’ development through time across both outcomes (short- and long-term SA): (i) determining the ideal number of trajectory groups for both sets of measurements, (ii) the probability of membership in each trajectory group and (iii) the probabilities that link membership in trajectory groups across the two measured phenomena.^22^ The model specifies both trajectories at the same time and the maximum posterior probability assigns participants to the trajectory group with the highest posterior probability in each trajectory model.^29^

The annual number of short-term SA spells and the annual number of long-term SA months were used as outcomes with a zero-inflated Poisson distribution given that during multiple years the participants had zero SA spells or no long-term SA. First, both SA outcomes were modelled independently. Models were tried with 1–7 trajectory groups and up to third-order polynomials. The number of trajectory groups was based on Bayesian Information Criterion (BIC), Akaike Information Criteria (AIC), entropy, and preference for clinically plausible models that produced trajectory groups with no fewer than 5% of the total sample. A model with three trajectory groups was selected for both outcomes (Supplementary material).

The dual trajectory model was fitted using starting values from the two models. Those with more SA were more likely to drop-out during the follow-up. To account for this non-random attrition, we modelled dropout-probability with dependence on two previous responses.^30^ We selected a dual model with three trajectories for both SA outcomes (Supplementary material). Mean posterior probabilities were >0.90, indicating a good model fit and a low risk of false classification.

Information on the trajectory group memberships was combined with survey and register data. The association of social-, work- and health-related factors with trajectory group membership was examined using Chi-square tests. We additionally fitted a multinomial logistic regression model of the trajectory group memberships and work-related factors. The trajectory analyses were computed with Stata 17 software’s traj-command. R was used for other analyses.

The ethics committees of the Department of Public Health, the University of Helsinki (decision 30 November 1998) and the health authorities of the City of Helsinki (decision 5 October 1999) have approved the Helsinki Health Study.

## Results

Routine non-manual and manual employees were the largest occupational class, accounting for half of the employees (table 1). Healthcare and social sectors were the two largest occupational sectors, accounting for two-thirds of the employees. One-fifth of the employees reported shift work, smoking and sleep problems, and two-thirds were cohabiting. One-fifth reported low leisure-time physical activity.

**Table 1:** Characteristics of the study population: sample of women employed at the city of Helsinki aged 46–55 years during years 2000–17 (*N* = 3206) and the determinants of sickness absence (SA) trajectory groups; Panel A: short-term SA model. Panel B: long-term SA model

	Study population	Panel A: Short-term SA trajectory group				Panel B: Long-term SA trajectory group			
		No short-term SA, *N* (%)	Low frequency short-term SA, *N* (%)	High frequency short-term SA, *N* (%)	*P*-value for Chi^2^	No long-term SA, *N* (%)	Low long-term SA, *N* (%)	High long-term SA, *N* (%)	*P*-value for Chi^2^
All		1607 (50)	1280 (40)	319 (10)		2139 (65)	818 (27)	254 (8)	
Occupational class									
Professionals and managers	858 (27)	555 (35)	258 (20)	45 (14)		690 (32)	142 (17)	26 (10)	
Semi-professionals	689 (21)	357 (22)	288 (23)	44 (14)		483 (23)	162 (20)	44 (17)	
Routine non-manual and manual workers	1658 (52)	693 (43)	737 (57)	228 (72)	< 0.001	959 (45)	514 (63)	185 (73)	< 0.001
Employment sector									
Teaching	463 (15)	283(18)	147 (11)	33 (10)		373 (18)	76 (9)	14 (6)	
Social	1306 (41)	562 (36)	586 (46)	158 (50)		818 (39)	373 (46)	115 (46)	
Healthcare	838 (26)	409 (26)	343 (27)	86 (27)		526 (25)	236 (29)	76 (30)	
Other	567 (18)	323 (20)	203 (16)	41 (13)	< 0.001	394 (19)	127 (16)	46 (18)	< 0.001
Marital status									
Cohabiting	2175 (68)	1127 (70)	868(68)	180 (56)		1507 (71)	527 (64)	141 (56)	
Non-cohabiting	1030 (32)	479 (30)	414 (32)	137 (44)	< 0.001	626 (29)	291 (36)	113 (44)	< 0.001
Working arrangements									
Non-shift work	2609 (81)	1344 (84)	1023 (80)	242 (76)		1785 (84)	643 (79)	181 (72)	
Shift work	595 (19)	260 (16)	260 (20)	75 (24)	0.0014	347 (16)	174 (21)	74 (28)	< 0.001
Work–family satisfaction									
Satisfied	1777 (56)	935 (59)	692 (54)	150 (49)		1230 (58)	430 (53)	117 (48)	
Dissatisfied	1384 (44)	646 (41)	581 (46)	157 (51)	0.0012	877 (42)	378 (47)	129 (52)	0.0010
Smoking									
Never or quit	2552 (80)	1377 (86)	969 (76)	206 (65)		1779 (83)	603(74)	170 (67)	
Daily or occasionally	652 (20)	229 (14)	312 (24)	111 (35)	< 0.001	353 (17)	214 (26)	85 (33)	< 0.001
BMI									
Healthy weight (BMI<25)	1642 (51)	913 (57)	600 (47)	129 (40)		1198 (56)	354 (43)	90 (35)	
Overweight (25≤BMI<30)	1001 (31)	488 (30)	423 (33)	90 (28)		639 (30)	273 (33)	89 (35)	
Obesity (BMI≥30)	561 (18)	204 (13)	259 (20)	98 (31)	< 0.001	296 (14)	190 (23)	75 (30)	< 0.001
Leisure-time physical activity									
High	1131 (35)	657 (41)	396 (31)	78 (25)		864 (41)	217 (27)	50 (20)	
Intermediate	1356 (42)	626 (39)	572 (45)	158 (50)		851 (40)	389 (48)	116 (46)	
Low	704 (22)	314 (20)	311 (24)	79 (25)	< 0.001	409 (19)	209 (26)	86 (35)	< 0.001
Sleep problems									
No	2507 (79)	1331 (83)	965 (76)	211 (67)		1749 (82)	607 (75)	151 (59)	
Yes	679 (21)	264 (17)	312 (24)	103 (33)	< 0.001	369 (18)	207 (25)	103 (41)	< 0.001

Notes: Values express *N* (%). SA, sickness absence; BMI, body mass index.

**Table 2:** The linkage between short-term and long-term SA trajectory groups presented by conditional and joint probabilities from the group-based dual trajectory model

Probability of long-term SA trajectory group (*k*) conditional on short-term SA trajectory group (*j*) [π_*k*__|__*j*_]				
Long-term SA trajectory group	Short-term SA trajectory group			
	No short-term SA (50%)	Low frequency short-term SA (40%)	High frequency short-term SA (10%)	
No long-term SA (65%)	0.85	0.5	0.24	
Low long-term SA (27%)	0.12	0.39	0.53	
High long-term SA (8%)	0.03	0.11	0.23	
	1	1	1	

Probability of short-term SA trajectory group (*j*) conditional on long-term SA trajectory group (*k*) [π_*j*__|__*k*_]				
Long-term SA trajectory group	Short-term SA trajectory group			
	No short-term SA (50%)	Low frequency short-term SA (40%)	High frequency short-term SA (10%)	
No long-term SA (65%)	0.66	0.31	0.04	1
Low long-term SA (27%)	0.22	0.58	0.20	1
High long-term SA (8%)	0.20	0.52	0.28	1

Joint probability of short-term SA trajectory groups (*j*) and long-term SA trajectory groups (*k*) [π_*kj*_]				
Long-term SA trajectory group	Short-term SA trajectory group			
	No short-term SA (50%)	Low frequency short-term SA (40%)	High frequency short-term SA (10%)	
No long-term SA (65%)	0.43	0.20	0.02	
Low long-term SA (27%)	0.06	0.16	0.05	
High long-term SA (8%)	0.02	0.04	0.02	
				1

Notes: Conditional probability describes how likely an individual is to be in each long-term SA trajectory group if a person is known to be in a given short-term SA trajectory group. Joint probability describes how likely overall it is for a person to fall into any combination of the short- and long-term SA trajectory groups. Joint probability membership is presented for every trajectory group combination, nine joint probabilities, that sum up to one. The names express the trajectory groups’ name and the percentage (%). SA, sickness absence.

### Short-term SA trajectories

**Figure 1:**
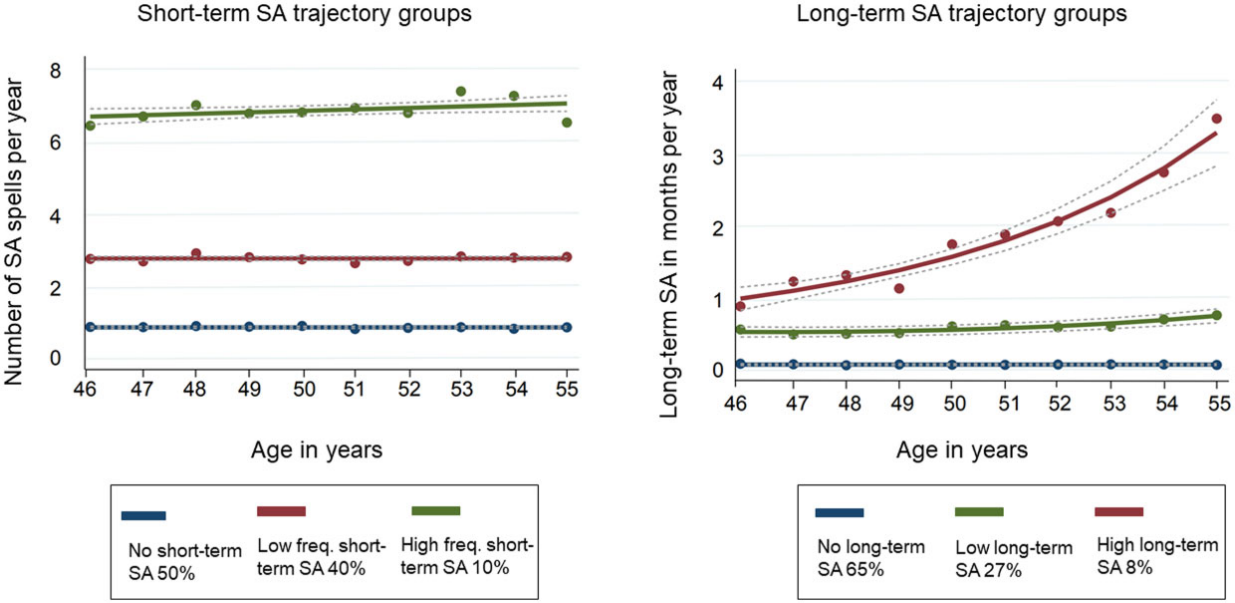
The developmental trajectories of sickness absence (SA) of employed women at the city of Helsinki between ages 46–55 (*N* = 3206). Left panel: The developmental trajectories for the number of short-term SA spells per year during ages 46–55: ‘no short-term SA’ (50%), ‘low frequency short-term SA’ (40%) and ‘high frequency short-term SA’ (10%). Right panel: The developmental trajectories of the number of long-term SA months per year during ages 46–55: ‘no long-term SA’ (65%), ‘low long-term SA’ (27%), ‘high long-term SA’ (8%). SA, sickness absence; freq., frequency

For short-term SA, we selected a model with three trajectory groups (figure 1). Two trajectory groups with the fewest spells—‘no short-term SA’ (50%, around 1 spell/year) and ‘low frequency short-term SA’ (40%, around 3 spells/year)—comprised 90% of the population. The third group, ‘high frequency short-term SA’, comprised 10% of the population and was well-separated from the two other groups by having a higher frequency of SA spells (around 7 spells/year). Trajectory groups describe the number of short-term SA spells in relation to time (here, age). No considerable change with age was noticed in the frequency of short-term SA spells in any of the trajectory groups (Supplementary material for individual trajectories).

### Long-term SA trajectories

For long-term SA, we selected a model with three trajectory groups (figure 1). Employees in the largest group, ‘no long-term SA’ (65%), had no long-term SA. Individuals in the second largest group, ‘low long-term SA’ (27%), had on average 2–3 weeks of SA with a gently increasing trend. Individuals in the smallest group, ‘high long-term SA’ (8%), had an increasing trend with SA rising from one month to four months per year during follow-up.

### Relationship between the SA trajectories

The dual trajectory model output produces a summary on the interrelationship of the outcomes (table 2). Regarding long-term SA conditional on short-term SA, those assigned to the trajectory group ‘no short-term SA’ had an 85% probability of being assigned to the ‘no long-term SA’ trajectory group and only 3% to ‘high long-term SA’, meaning that there exists a clear interrelationship with the trajectory groups as employees taking no short-term SA were very likely to follow a path without SA in the long-term SA model. In contrast, when assigned to the trajectory group ‘high frequency short-term SA’ the same probabilities were 24% and 23%, respectively. This embodies uncertainty in the long-term SA trajectories of employees who take multiple short-term SA spells. This is denoted by the equal probability of following either the trajectory with no long-term SA (24%) or the trajectory with high long-term SA (23%), while there is still a 53% probability of following a path with low long-term SA.

Second, looking at short-term SA conditional on the long-term SA trajectory group, those assigned to the ‘no long-term SA’ trajectory group had a 66% probability of being assigned to ‘no short-term SA’ and only 4% probability of being assigned to the ‘high frequency short-term SA’ trajectory group. When assigned to the ‘high long-term SA’ trajectory group, the probabilities were 20% and 28%, respectively. That is, the probability of belonging to any specific short-term SA group when assigned to the ‘high long-term SA’ trajectory group was far less certain, implying population heterogeneity in the developmental course of SA.

Lastly, the joint probability memberships show a fair amount of overlap but also unexpected developmental patterns. Having more short-term SA spells is linked to a higher possibility of more long-term SA, but the trajectories do not always develop in the same direction; those having frequent short-term SA spells do not always end up with high long-term SA and not all participants leading to the ‘high long-term SA’ trajectory group could have been foretold by their short-term SA patterns.

### Determinants of trajectory group membership

Employees in the lowest occupational class were more prevalent in the trajectories with high SA in both SA models (table 1). The healthcare and social sectors showed increased prevalence in trajectory groups with high SA. Similarly, shift work, being dissatisfied in combining paid work with family life, and non-cohabiting were more prevalent in the trajectory groups with low or high short- or long-term SA. Regarding health-related factors, smoking, sleep problems, higher BMI and low leisure-time physical activity were more prevalent in the low and high SA trajectory groups than no SA trajectory groups. The differences were clearer in the long-term SA model.

Differences between employment sectors remained when adjusting for occupational class and work type in the multinomial logistic regression model for trajectory group membership and work-related factors (Supplementary table). The social sector was associated with a higher likelihood of being assigned to ‘low frequency short-term SA’ and ‘high frequency short-term SA’ trajectory groups and healthcare to the ‘high frequency short-term SA’ trajectory group in the short-term SA model. In the long-term SA model, the healthcare and social sectors were associated with a higher likelihood of being assigned to the ‘high long-term SA’ trajectory group compared with employment sector ‘other’.

### Sensitivity analysis

Attrition during follow-up was highest in the ‘high long-term SA’ trajectory group (median follow-up 7 years). This reflects the general idea of long-term SA preceding disability pension and the legislation in Finland according to which an employee must have been on long-term SA prior being granted disability pension. We tested if the trajectories would change when analysing shorter time periods. The short-term model produced similar trajectories when analysed separately during ages 46–50 and 51–55. The long-term model produced similar trajectories in shape, but the high long-term SA trajectory was around 3% smaller. Hypothetically, fewer employees had time to move to disability pension during a shorter period. We tested the model with both genders and the trajectories were not significantly altered.

## Discussion

We discovered that SA rates did not increase with age among most employees. The number of short-term SA spells did not increase with age and only a tenth of the employees were assigned to a high frequency short-term SA trajectory. A third of the employees had some increase in long-term SA with age, but only 8% of all the employees were assigned to a trajectory group with notably increasing long-term SA. The short- and long-term SA trajectories were interconnected, and population heterogeneity was noticed. The descriptive results were as expected; lower occupational class and poorer health behaviours were associated with the trajectory groups with more SA. Employees in healthcare and social sectors were more likely to be assigned to a high SA trajectory group even when adjusting for occupational class and work arrangements.

Few previous studies have examined SA trajectories among women. A Spanish study identified three trajectories with most employed women being assigned to a low stable trajectory,^31^ the percentage of which is in line with our results. A Finnish study examined the combined short- and long-term SA trajectories of municipal and private sector employees among both men and women, and the smallest group with the most SA had around 60 days absence per year, which increased only slightly during follow-up.^32^ This is roughly the mean SA length in our trajectory group ‘high long-term SA’. They, however, used calendar years, whereas we examined SA with age using a longer follow-up.

Stability in the number of short-term SA spells with age reflects the general understanding of SA; short-term SA is either self-certified or administered due to minor diseases or in the beginning of a more severe disease and short-term SA spells are more associated with motivational factors.^33^ However, not only individual differences, but also occupational class differences are likely to explain this pattern, as those in higher occupational classes might more easily be able to work while sick and have better possibilities to modify working tasks. In the smaller subgroups with high short-term SA, SA could perhaps be reduced by targeting work arrangements and motivational factors. If so, the frequency of SA spells would be utilizable in screening while considering that the developmental patterns of short-term SA show heterogeneity in the long-term SA patterns.

Consistent occupational class differences in SA were found. Over 70% of the participants assigned to high SA trajectory groups were routine non-manual employees and manual workers while this occupational class accounts for half of the study population. Especially long-term SA is strongly associated with health and these findings reflect on the general health disparities between occupational classes.

A recent Swedish study found that employees in female-dominated workplaces are at higher risk of SA compared with gender-equal or male-dominated workplaces.^34^ Our findings suggest a similar pattern in our data, though limited to only one large public sector employer in Finland. Two occupational sectors where the majority of employees are women—healthcare and social—appeared more prone to prolonging SA or more frequent short-term SA spells. Reasons for this can be speculated; job descriptions in healthcare and social sectors are both mentally and physically demanding, and employees might do shift work or suffer from infectious diseases. In addition, staff shortages or ethical dilemmas might affect employees’ motivation and increase the likelihood of SA. On the other hand, no SA differences were noticed in the employment sector teaching, which is also a sector with more women and contact with infectious diseases. Differences might exist in absence culture or in the use of SA to cope with work-related strain.

Multiple work- and health-related factors have been linked to a higher risk of short- or long-term SA and prolonging absence among women, and our results correspond to that. Among nurses, working arrangements such as night shift work have been associated with long-term SA in Denmark and Finland,^35^ and long weekly working hours with short-term SA^36^ in Finland. Our results with a novel method support the previous well-established association of health-related factors and SA.^3^ The predictors of short- and long-term SA were largely similar, with those reporting smoking, low leisure-time physical activity, poorer sleep and higher BMI being more prevalent in groups with higher SA rates. More adverse health-related factors and SA accumulate among a minority of employees.

The double burden hypothesis has been proposed as the explanation for women’s higher SA prevalence.^18^^,^^19^ A previous review suggests measuring experienced family stressors instead of the number of children as a better tool to examine this burden of multiple roles^37^ and previous studies have reported an association between work–family conflict and health.^38^ Our results support these findings, those with more SA more often replied being dissatisfied with combining paid work and family.

## Strengths and limitations

Strengths of the study included a large cohort data, a novel method to study SA patterns and the possibility to include four well-documented health-related risk factors for SA.^3^^,^^39^

The group-based dual trajectory model produces an informative picture of SA development; however, trajectories are always an approximation of reality. The model divides the population into distinctive trajectories with as much similar developmental features as possible. Trajectory group membership is assigned according to which group the individual has the highest posterior probability of belonging to. Conclusions on the trajectories’ determinants should therefore be made with care. The dual model’s conditional and joint probabilities provide information on the distribution of probabilities and heterogenic continuity, but we cannot assess causality.

We analysed SA development among employees in midlife, thus selecting those healthy enough to stay in the work force. The healthy worker effect should therefore be considered, meaning that people with a diminished work ability might have been selected out of the population prior to our study.^40^ Exiting the study was also more likely among employees with more long-term SA. We did not have the specific diagnostic codes for the SA. We have, however, analysed the most common diagnosis-specific long-term SA trajectories in our previous publication.^20^

## Conclusions

We discovered that the development of short- and long-term SA is interconnected but that these outcomes do not always develop in the same direction. SA does not increase among most ageing employees, but when the SA rate is high, SA develops in varying ways. Adverse health-related factors appear as significant determinants for both short- and long-term SA, and there were differences in proneness to SA between employment sectors.

## Supplementary Material

ckae023_Supplementary_Data

## Data Availability

The Helsinki Health Study survey data and the register data cannot be made publicly available due data protection laws. The survey data can only be used for scientific research by the research group. Collaboration requests should be sent to the principal investigators. Sickness absence (SA) increased only among a minority of women. The short-term SA spell trajectories remained stable with age, whereas the length of absence increased with age in two of the long-term SA trajectories. The short-term and long-term SA trajectories were interconnected, and population heterogeneity was noticed. Two large occupational sectors—healthcare and social—had a higher probability of more frequent short-term SA spells and prolonging to long-term SA, even when adjusting for other work-related factors.

## References

[1] European Foundation for the Improvement of Living and Working Conditions. *Absence from Work*. Dublin: European Foundation for the Improvement of Living and Working Conditions, 2010.

[2] Pekkala J , Blomgren J, Pietiläinen O, et al Occupational class differences in diagnostic-specific sickness absence: a register-based study in the Finnish population, 2005-2014. BMC Public Health 2017;17:670. 28830389 10.1186/s12889-017-4674-0 PMC5568169

[3] Virtanen M , Ervasti J, Head J, et al Lifestyle factors and risk of sickness absence from work: a multicohort study. Lancet Public Health 2018;13:e545–54. 10.1016/S2468-2667(18)30201-9 PMC6220357 30409406

[4] Merkus SL , van Drongelen A, Holte KA, et al The association between shift work and sick leave: a systematic review. Occup Environ Med 2012;69:701–12. 22767871 10.1136/oemed-2011-100488 PMC3597215

[5] Dekkers-Sanchez P , Hoving J, Sluiter J, Frings-Dresen M. Factors associated with long-term sick leave in sick-listed employees: a systematic review. Occup Environ Med 2008;65:153–7. 17881466 10.1136/oem.2007.034983

[6] Kristensen TS. Sickness absence and work strain among Danish slaughterhouse workers: an analysis of absence from work regarded as coping behaviour. Soc Sci Med 1991;32:15–27. 2008617 10.1016/0277-9536(91)90122-s

[7] Leinonen T , Laaksonen M, Chandola T, Martikainen P. Health as a predictor of early retirement before and after introduction of a flexible statutory pension age in Finland. Soc Sci Med 2016;158:149–57. 27155163 10.1016/j.socscimed.2016.04.029

[8] Khan J , Rehnberg C. Perceived job security and sickness absence: a study on moral hazard. Eur J Health Econ 2009;10:421–8. 19283417 10.1007/s10198-009-0146-5

[9] Feeney A , North F, Head J, et al Socioeconomic and sex differentials in reason for sickness absence from the Whitehall II Study. Occup Environ Med 1998;55:91–8. 9614392 10.1136/oem.55.2.91 PMC1757555

[10] Harkko J , Nordquist H, Pietiläinen O, et al Frequent short sickness absence, occupational health service utilisation and long-term sickness absence due to mental disorders among young employees. Int Arch Occup Environ Health 2021;94:1549–58. 34095973 10.1007/s00420-021-01728-5 PMC8384820

[11] Laaksonen M , He L, Pitkäniemi J. The durations of past sickness absences predict future absence episodes. J Occup Environ Med 2013;55:87–92. 23235465 10.1097/JOM.0b013e318270d724

[12] Labriola M , Lund T. Self-reported sickness absence as a risk marker of future disability pension. Prospective findings from the DWECS/DREAM study 1990-2004. Int J Med Sci 2007;4:153–8. 17554400 10.7150/ijms.4.153 PMC1885553

[13] Kivimäki M , Head J, Ferrie JE, et al Sickness absence as a prognostic marker for common chronic conditions: analysis of mortality in the GAZEL study. Occup Environ Med 2008; 165:820–6. 18611969 10.1136/oem.2007.038398 PMC2715845

[14] Kivimäki M , Head J, Ferrie JE, et al Sickness absence as a global measure of health: evidence from mortality in the Whitehall II prospective cohort study. BMJ 2003; 327:364. 12919985 10.1136/bmj.327.7411.364 PMC175810

[15] Gjesdal S , Bratberg E. Diagnosis and duration of sickness absence as predictors for disability pension: results from a three-year, multi-register based and prospective study. Scand J Public Health 2003;31:246–54. 15099029 10.1080/14034940210165154

[16] Marmot M , Feeney A, Shipley M, et al Sickness absence as a measure of health status and functioning: from the UK Whitehall II study. J Epidemiol Community Health 1995;49:124–30. 7798038 10.1136/jech.49.2.124 PMC1060095

[17] Lallukka T , Haaramo P, Rahkonen O, Sivertsen B. Joint associations of sleep duration and insomnia symptoms with subsequent sickness absence: the Helsinki Health Study. Scand J Public Health 2013;41:516–23. 23520224 10.1177/1403494813481647

[18] Laaksonen M , Mastekaasa A, Martikainen P, et al Gender differences in sickness absence – the contribution of occupation and workplace. Scand J Work Environ Health 2010;36:394–403. 20213051 10.5271/sjweh.2909

[19] Mastekaasa A. The gender gap in sickness absence: long-term trends in eight European countries. Eur J Public Health 2014;24:656–62. 24903105 10.1093/eurpub/cku075

[20] Suur-Uski J , Pietiläinen O, Salonsalmi A, et al Long-term sickness absence trajectories among ageing municipal employees – the contribution of social and health-related factors. BMC Public Health 2023;23:1429. 37495983 10.1186/s12889-023-16345-9 PMC10373243

[21] Stapelfeldt CM , Nielsen CV, Andersen NT, et al Sick leave patterns as predictors of disability pension or long-term sick leave: a 6.75-year follow-up study in municipal eldercare workers. BMJ Open 2014;4:e003941. 10.1136/bmjopen-2013-003941 PMC3918999 24508850

[22] Nagin D. Group-Based Modeling of Development. Cambridge: Harvard University Press, 2005. Available at: http://ebookcentral.proquest.com/lib/helsinki-ebooks/detail.action?docID=3300109 (12 December 2023, date last accessed).

[23] Lahelma E , Aittomäki A, Laaksonen M, et al Cohort profile: the Helsinki Health Study. Int J Epidemiol 2013;42:722–30. 22467288 10.1093/ije/dys039

[24] Wallman T , Wedel H, Palmer E, et al Sick-leave track record and other potential predictors of a disability pension. A population based study of 8,218 men and women followed for 16 years. BMC Public Health 2009;9:104. 19368715 10.1186/1471-2458-9-104 PMC2674437

[25] Lahelma E , Martikainen P, Rahkonen O, et al Occupational class inequalities across key domains of health: results from the Helsinki Health Study. Eur J Public Health 2005;115:504–10. 16014660 10.1093/eurpub/cki022

[26] Ainsworth B , Haskell W, Herrmann S, et al 2011 Compendium of Physical Activities: a second update of codes and MET values. Med Sci Sports Exerc 2011;43:1575–81. 21681120 10.1249/MSS.0b013e31821ece12

[27] Jenkins CD , Stanton BA, Niemcryk SJ, Rose RM. A scale for the estimation of sleep problems in clinical research. J Clin Epidemiol 1988;41:313–21. 3351539 10.1016/0895-4356(88)90138-2

[28] Lallukka T , Dregan A, Armstrong D. Comparison of a sleep item from the General Health Questionnaire-12 with the Jenkins Sleep Questionnaire as measures of sleep disturbance. J Epidemiol 2011;21:474–80. 21986193 10.2188/jea.JE20110023 PMC3899464

[29] Graziane JA , Beer JC, Snitz BE, et al Dual trajectories of depression and cognition: a longitudinal population-based study. Am J Geriatr Psychiatry 2016;24:364–73. 26560510 10.1016/j.jagp.2015.08.001 PMC4841743

[30] Haviland AM , Jones BL, Nagin DS. Group-based trajectory modeling extended to account for nonrandom participant attrition. Sociol Methods Res 2011;40:367–90.

[31] Ubalde-Lopez M , Hernando-Rodriguez JC, Benavides FG, Serra L. Trajectories of sickness absence among salaried workers: evidence from the WORKss cohort in Catalonia (Spain), 2012-2014. BMJ Open 2019;9:e029092. 10.1136/bmjopen-2019-029092 PMC6615827 31272980

[32] Virtanen P , Siukola A, Lipiäinen L, et al Trajectory analyses of sickness absence among industrial and municipal employees. Occup Med (Lond) 2017;67:109–13. 27496546 10.1093/occmed/kqw104

[33] Janssen N , Kant IJ, Swaen GM, et al Fatigue as a predictor of sickness absence: results from the Maastricht cohort study on fatigue at work. Occup Environ Med 2003;60 Suppl 1:i71–6. 12782750 10.1136/oem.60.suppl_1.i71 PMC1765725

[34] Haukenes I , Hammarström A. Workplace gender composition and sickness absence: a register-based study from Sweden. Scand J Public Health 2023;0(0).14034948231176108. 37265198 10.1177/14034948231176108 PMC11308254

[35] Larsen AD , Ropponen A, Hansen J, et al Working time characteristics and long-term sickness absence among Danish and Finnish nurses: a register-based study. Int J Nurs Stud 2020;112:103639. 32505388 10.1016/j.ijnurstu.2020.103639

[36] Ropponen A , Koskinen A, Puttonen S, Härmä M. Exposure to working-hour characteristics and short sickness absence in hospital workers: a case-crossover study using objective data. Int J Nurs Stud 2019;91:14–21. 30665013 10.1016/j.ijnurstu.2018.11.002

[37] Nilsen W , Skipstein A, Østby KA, Mykletun A. Examination of the double burden hypothesis—a systematic review of work–family conflict and sickness absence. Eur J Public Health 2017;27:465–71. 28486653 10.1093/eurpub/ckx054 PMC5445721

[38] Borgmann LS , Rattay P, Lampert T. Health-related consequences of work-family conflict from a European perspective: results of a scoping review. Front Public Health 2019;7:189. 31338358 10.3389/fpubh.2019.00189 PMC6629821

[39] Kanerva N , Lallukka T, Rahkonen O, et al The joint contribution of physical activity, insomnia symptoms, and smoking to the cost of short-term sickness absence. Scand J Med Sci Sports 2019;29:440–9. 30480836 10.1111/sms.13347

[40] Wilcosky T , Wing S. The healthy worker effect. Selection of workers and work forces. Scand J Work Environ Health 1987;13:70–2. 3576148 10.5271/sjweh.2078

