# Supplementary material for: Dual trajectories of short-term and long-term sickness absence and their social- and health-related determinants among women in the public sector
Source: Eur J Public Health. 2024 Feb 20;34(2):322–8. doi: 10.1093/eurpub/ckae023 (PMC10990561; doi:10.1093/eurpub/ckae023)
Supplement: ckae023_Supplementary_Data [file ckae023_supplementary_data.docx]

Dual Trajectories of Short-term and Long-term Sickness Absence and their Social- and Health-related Determinants among Women in the Municipal Sector.

Supplementary material

## **Supplementary Table 1:** Syntax used for the final dual model

traj, var(sh*) indep(t*) model(zip) order(0 0 1) dropout (2 2 2) ^a^ var2(snmln*) indep2(t*) model2(zip) order2(0 2 2) dropout^a^ (2 2 2) detail start(strt)

^a^ dropout(2 2 2) include logistic model of dropout probability per wave. For each group, 2 = depends on the two previous responses

## **Supplementary Table 2:** Short-term SA outcome, inspecting the model with varying number of trajectory groups with three-order polynomials

| Number of classes presented by the number of digits in order with 3 meaning cubical | | % of trajectory groups |
| --- | --- | --- |
| traj, var(sh*) indep(t*) model(zip) order(3) detail | BIC=-57198.12 (N=24747) BIC=-57194.03 (N=3206) AIC=-57181.89 ll= -57177.89 | /100 |
| traj, var(sh*) indep(t*) model(zip) order(3 3) detail | BIC=-46852.64 (N=24747) BIC=-46843.44 (N=3206) AIC=-46816.11 ll= -46807.11 | 70/30 |
| traj, var(sh*) indep(t*) model(zip) order(3 3 3) detail | BIC=-44314.40 (N=24747) BIC=-44300.09 (N=3206) AIC=-44257.59 ll= -44243.59 | 44/43/11 |
| traj, var(sh*) indep(t*) model(zip) order(3 3 3 3) detail | BIC=-43663.66 (N=24747) BIC=-43644.24 (N=3206) AIC=-43586.55 ll= -43567.55 | 38/42/16/3 |
| traj, var(sh*) indep(t*) model(zip) order(3 3 3 3) detail | BIC=-43304.74 (N=24747) BIC=-43280.22 (N=3206) AIC=-43207.35 ll= -43183.35 | 23/37/27/10/1 |
| traj, var(sh*) indep(t*) model(zip) order(3 3 3 3 3 3) detail | BIC=-43320.60 (N=24747) BIC=-43290.96 (N=3206) AIC=-43202.91 ll= -43173.91 | 23/27/37/10/1/0.3 |
| traj, var(sh*) indep(t*) model(zip) order(3 3 3 3 3 3 3) detail | BIC=-43210.83 (N=24747) BIC=-43176.09 (N=3206) AIC=-43072.85 ll= -43038.85 | 12/29/18/30/8/1.5/0.2 |

## **Supplementary Table 3:** Determining polynomials for short-term SA model with three trajectory groups while accounting for non-random attrition

| traj, var(sh*) indep(t*) model(zip) order(3 3 3) dropout (2 2 2) detail | BIC=-46214.79 (N=24747) BIC=-46191.29 (N=3206) AIC=-46121.45 ll= -46098.45 | entropy: 0.878 |
| --- | --- | --- |
| traj, var(sh*) indep(t*) model(zip) order(3 3 3) dropout (2 2 2) detail start(strt) | BIC=-46214.79 (N=24747) BIC=-46191.29 (N=3206) AIC=-46121.45 ll= -46098.45 | entropy: 0.878 |
| . traj, var(sh*) indep(t*) model(zip) order(2 2 2) dropout (2 2 2) detail start(strt) | BIC=-46202.52 (N=24747) BIC=-46182.09 (N=3206) AIC=-46121.36 ll= -46101.36 | Entropy = 0.877 |
| traj, var(sh*) indep(t*) model(zip) order(2 1 1) dropout (2 2 2) detail start(strt) | BIC=-46196.05 (N=24747) BIC=-46177.66 (N=3206) AIC=-46123.00 ll= -46105.00 | 0.877 |
| traj, var(sh*) indep(t*) model(zip) order(1 1 1) dropout (2 2 2) detail start(strt) | BIC=-46191.00 (N=24747) BIC=-46173.63 (N=3206) AIC=-46122.01 ll= -46105.01 | 0.877 |
| . traj, var(sh*) indep(t*) model(zip) order(0 1 1) dropout (2 2 2) detail start(strt) | BIC=-46185.97 (N=24747) BIC=-46169.62 (N=3206) AIC=-46121.04 ll= -46105.04 | 0.877 |
| traj, var(sh*) indep(t*) model(zip) order(0 0 1) dropout (2 2 2) detail start(strt) | BIC=-46183.60 (N=24747) BIC=-46168.27 (N=3206) AIC=-46122.72 ll= -46107.72 | 0.877 |
| 0 0 1 chosen |  | Figure below |

## **Supplementary Figure** Individual trajectories, spaghetti plot of short-term SA by trajectory groups

## **Supplementary Table 4:** Long-term SA outcome: inspecting the model with varying number of trajectory groups with three-order polynomials

| Number of classes presented by the number of digits in order with 3 meaning cubical | | Entropy | % of trajectory groups |
| --- | --- | --- | --- |
|  |  |  |  |
| traj, var( snmln*) indep(t*) model(zip) order(3) detail | BIC=-21860.50 (N=24747) BIC=-21856.41 (N=3206) AIC=-21844.26 ll= -21840.26 | | 100 |
| traj, var(snmln*) indep(t*) model(zip) order(3 3) detail | BIC=-18210.28 (N=24747) BIC=-18201.09 (N=3206) AIC=-18173.76 ll= -18164.76 | Entropy = 0.876 | 77/23 |
| traj, var(snmln*) indep(t*) model(zip) order(3 3 3) detail | BIC=-17687.35 (N=24747) BIC=-17673.05 (N=3206) AIC=-17630.54 ll= -17616.54 | Entropy = 0.805 | 62/31/7 |
| traj, var(snmln*) indep(t*) model(zip) order(3 3 3 3) detail | BIC=-17276.42 (N=24747) BIC=-17257.01 (N=3206) AIC=-17199.31 ll= -17180.31 | Entropy = 0.813 | 10/64/21/5 |
| traj, var(snmln*) indep(t*) model(zip) order(3 3 3 3 3) detail | BIC=-17054.42 (N=24747) BIC=-17029.90 (N=3206) AIC=-16957.03 ll= -16933.03 | Entropy = 0.799 | 12/612/9/13/4 |
| traj, var(snmln*) indep(t*) model(zip) order(3 3 3 3 3 3) detail | BIC=-16917.95 (N=24747) BIC=-16888.32 (N=3206) AIC=-16800.26 ll= -16771.26 | Entropy = 0.788 | 8/11/58/4/16/2 |
| traj, var(snmln*) indep(t*) model(zip) order(3 3 3 3 3 3 3) detail | BIC=-16836.61 (N=24747) BIC=-16801.87 (N=3206) AIC=-16698.63 ll= -16664.63 | Entropy = 0.735 | 12/9/50/16/2/5 |

## **Supplementary Table 5:** Choosing the order of polynomials for three trajectory group long-term SA model

| traj, var(snmln*) indep(t*) model(zip) order(3 3 3) dropout (2 2 2 2) detail | BIC=-19087.00 (N=24747) BIC=-19055.33 (N=3206) AIC=-18961.20 ll= -18930.20 | Entropy = 0.816 |
| --- | --- | --- |
| traj, var(snmln*) indep(t*) model(zip) order(3 3 3) dropout (2 2 2) detail start(strt) | BIC=-19493.68 (N=24747) BIC=-19470.18 (N=3206) AIC=-19400.34 ll= -19377.34 | Entropy = 0.804 |
| traj, var(snmln*) indep(t*) model(zip) order(2 2 3) dropout (2 2 2) detail start(strt) | BIC=-19485.04 (N=24747) BIC=-19463.58 (N=3206) AIC=-19399.81 ll= -19378.81 | Entropy = 0.803 |
| traj, var(snmln*) indep(t*) model(zip) order(1 2 3) dropout (2 2 2) detail start(strt) | BIC=-19480.13 (N=24747) BIC=-19459.70 (N=3206) AIC=-19398.97 ll= -19378.97 | Entropy = 0.803 |
| traj, var(snmln*) indep(t*) model(zip) order(1 2 2) dropout (2 2 2) detail start(strt) | BIC=-19475.32 (N=24747) BIC=-19455.90 (N=3206) AIC=-19398.21 ll= -19379.21 | Entropy = 0.804 |
| traj, var(snmln*) indep(t*) model(zip) order(0 2 2) dropout (2 2 2) detail start(strt) | BIC=-19472.48 (N=24747) BIC=-19454.09 (N=3206) AIC=-19399.43 ll= -19381.43 | Entropy = 0.804 |
| traj, var(snmln*) indep(t*) model(zip) order(0 1 2) dropout (2 2 2) detail start(strt) | BIC=-19478.72 (N=24747) BIC=-19461.35 (N=3206) AIC=-19409.73 ll= -19392.73 | Entropy = 0.807 |

Model with 0 2 2 results in best BIC

## **Supplementary Figure :** Individual trajectories, spaghetti plot of long-term SA by trajectory groups

##

**The Dual trajectory model output**

matrix strt = -0.18459, 1.00670, 1.89064, 0.00589, -3.84847, -0.23299, 0.20649, -4.41682, -0.04404, 0.18219, -3.89639, -0.05341, 0.10283, 50.09760, 39.85432, 10.04808, -2.78451, -0.12312, 0.09499, 0.00360, -0.65333, -0.01790, 0.00527, -4.07942, 0.22590, 0.44562, -2.38059, 0.26466, -0.14070, -19.28198, -1.26236, 0.69122, 85.04477, 3.31056, 11.64467, 49.92183, 10.79820, 39.27996, 23.60060, 23.45918, 52.94022

traj, var(sh*) indep(t*) model(zip) order(0 0 1) dropout (2 2 2) var2(snmln*) indep2(t*) model2(zip) order2(0 2 2) dropout (2 2 2) detail start(strt)

Short-term SA model and drop-out probabilities by trajectory group.

Long-term SA model and drop-out probabilities by trajectory group.

**Supplementary table 6:** Long-term SA model and drop-out probabilities by trajectory group.

**Average posterior probabilities:**

**Short-term SA model**

No short-term SA 0.96

Low frequency short-term SA 0.94

High frequency short-term SA 0.96

**Long-term SA model**

No long-term SA 0.95

Low long-term SA 0.90

High long-term SA 0.95

**Supplementary Table 7:** Multinomial logistic regression model occupational class + employment sector + work type

| Short-term SA model | Low frequency short-term SA | High frequency short-term SA |
| --- | --- | --- |
| Reference trajectory group No SA | OR (95% CI) | OR (95% CI) |
| Occupational class (reference managers and professionals) |  |  |
| Semi-professionals | 1.58 (1.24 to 2.01)* | 1.38 (0.86 to 2.22) |
| Routine non-manual workers | 2.10 (1.71 to 2.58)* | 3.75 (2.54 to 5.50)* |
| Field of occupation (reference other) |  |  |
| Teaching | 1.16 (0.87 to 1.53) | 1.67 (0.99 to 2.82) |
| Social | 1.53 (1.23 to 1.89)* | 2.06 (1.41 to 3.01)* |
| Health | 1.26 (0.99 to 1.59) | 1.69 (1.11 to 2.57)* |
| Worktype (reference daytime work) |  |  |
| Shiftwork | 1.12 (0.91 to 1.37) | 1.20 (0.87 to 1.65) |
| multinomial logistic regression trajectory groups - occupational class + field of occupation + work type |  |  |

OR= Odds ratio CI= confidence interval

| Long-term SA model | Low long-term SA | High long-term SA |
| --- | --- | --- |
| Reference trajectory group No SA | OR (95% CI) | OR (95% CI) |
| Occupational class (reference managers and professionals) |  |  |
| Semi-professionals | 1.80 ( 1.05 to 3.08)* | 1.32 (0.99 to 1.74) |
| Routine non-manual workers | 3.87 (2.44 to 6.13)* | 2.23 (1.76 to 2.82)* |
| Field of occupation (reference other) |  |  |
| Teaching | 0.63 (0.33 to 1.19) | 0.85 (0.61 to 1.20) |
| Social | 1.07 (0.74 to 1.56) | 1.33 (1.05 to 1.70)* |
| Health | 1.11 (0.74 to 1.68) | 1.40 (1.08 to 1.82)* |
| Worktype (reference daytime work) |  |  |
| Shiftwork | 1.54 (1.12 to 2.11)* | 1.07 (0.86 to 1.33) |
| multinomial logistic regression trajectory groups - occupational class + field of occupation + work type |  |  |

OR= Odds ratio CI= confidence interval
